# Supplementary material for: Establishing the carrier scattering phase diagram for ZrNiSn-based half-Heusler thermoelectric materials
Source: Nat Commun. 2020 Jun 19;11:3142. doi: 10.1038/s41467-020-16913-2 (PMC7305298; doi:10.1038/s41467-020-16913-2)
Supplement: Supplementary file 1 — Supplementary Information [file 41467_2020_16913_MOESM1_ESM.pdf]

Supplement Information

Establishing the carrier scattering phase diagram for ZrNiSn-based half-Heusler thermoelectric materials

Qingyong Ren[1], Chenguang Fu[2], Qinyi Qiu[3], Shengnan Dai[4], Zheyuan Liu[1], Takatsugu Masuda[5], Shinichiro Asai[5], Masato Hagihala[6], Sanghyun Lee[6], Shuki Torri[6], Takashi Kamiyama[6,7], Lunhua He[8,9,10], Xin Tong[10,11], Claudia Felser[2], David J. Singh[12], Tiejun Zhu[3], Jiong Yang[4], and Jie Ma[1,13]

[1]Key Laboratory of Artificial Structures and Quantum Control, School of Physics and Astronomy, Shanghai Jiao Tong University, 800 Dongchuan Road, Shanghai 200240, China
[2]Max Planck Institute for Chemical Physics of Solids, Nöthnitzer Straße 40, 01187 Dresden, Germany
[3]State Key Laboratory of Silicon Materials, School of Materials Science and Engineering, Zhejiang University, Hangzhou 310027, China
[4]Materials Genome Institute, Shanghai University, 99 Shangda Road, Shanghai 200444, China
[5]Neutron Science Laboratory, Institute for Solid State Physics, University of Tokyo, Kashiwanoha, Kashiwa, 277-8581, Japan
[6]Institute of Materials Structure Science, High Energy Accelerator Research Organization (KEK), Tokai, Ibaraki 319-1106, Japan
[7]Department of Materials Structure Science, Sokendai (The Graduate University for Advanced Studies), Tokai, Ibaraki 319-1106, Japan
[8]Beijing National Laboratory for Condensed Matter Physics, Institute of Physics, Chinese Academy of Sciences, Beijing 100190, China
[9]Songshan Lake Materials Laboratory, Dongguan, Guangdong 523808, China
[10]Spallation Neutron Source Science Center, Dongguan 523803, China
[11]Institute of High Energy Physics, Chinese Academy of Sciences, Beijing 100049, China
[12]Department of Chemistry and Department of Physics and Astronomy, University of Missouri-Columbia, Columbia, MO 65211, USA
[13]Shenyang National Laboratory for Materials Science, Institute of Metal Research, Chinese Academy of Sciences, Shenyang, 110016, China

Correspondence and requests for materials should be addressed to C.G.F. (email: Chenguang.Fu@cpfs.mpg.de) or to J.Y. (email: jiongy@t.shu.edu.cn) or to J.M. (email: jma3@sjtu.edu.cn).

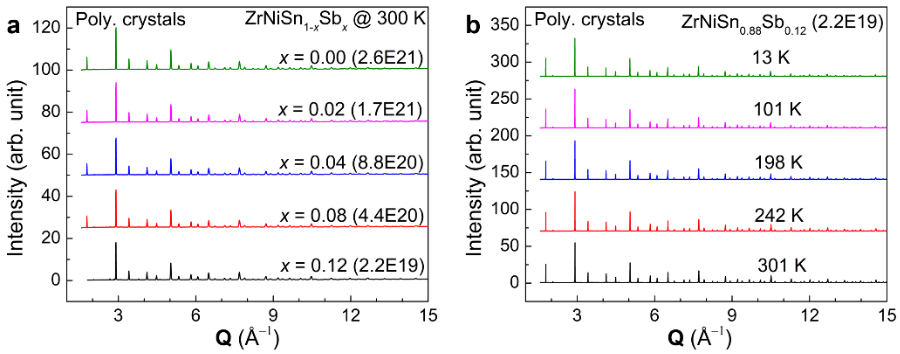

Supplementary Figure 1 Neutron powder diffraction patterns of (a) polycrystalline $ZrNiSn_{1-x}Sb_x$ with different carrier concentrations collected at 300 K on the GPPD, CSNS and of (b) $ZrNiSn_{0.88}Sb_{0.12}$ collected at different temperatures on the SuperHRPD, J-PARC. Rietveld refinements show that all the polycrystalline samples contain 5-7 % more Ni at the *4d* (¾, ¾, ¾) vacancy position, being consistent with the EPMA (electron probe microanalysis) analysis.[1]

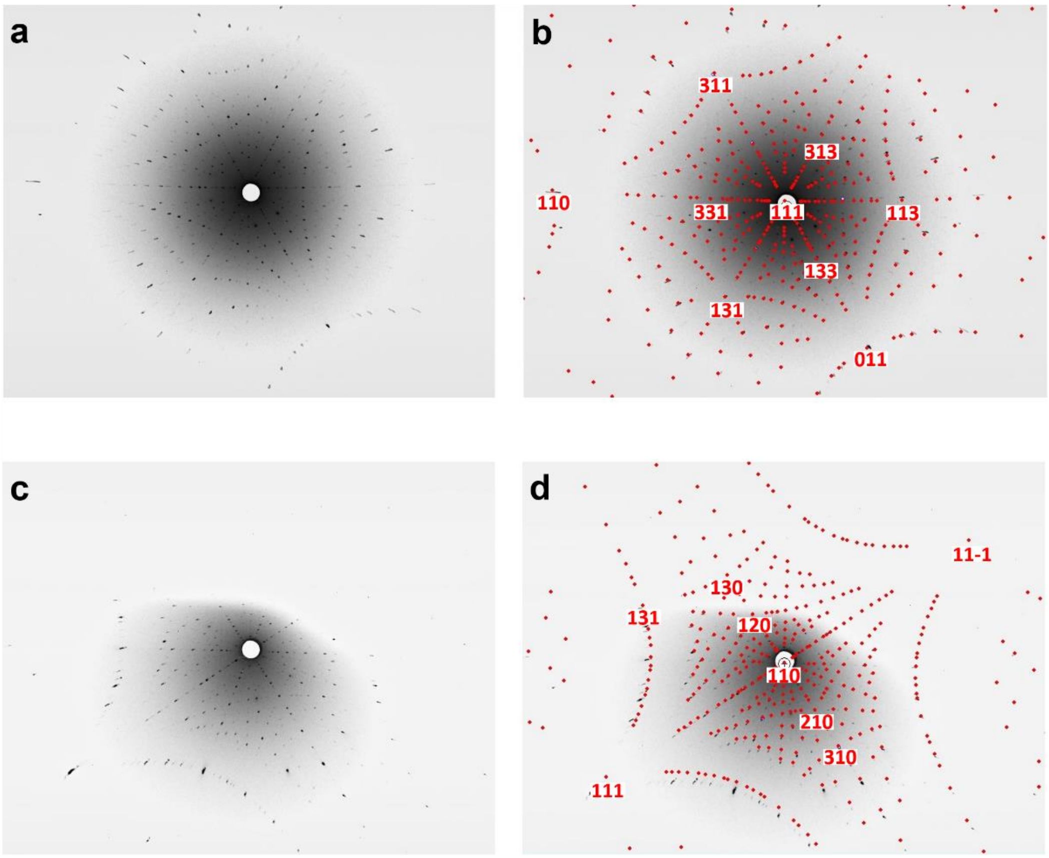

Supplementary Figure 2 Orientation of the single crystals with Laue diffraction. (a) The diffraction pattern for a typical (111) surface with the simulated pattern and Miller indices in (b). (c) The diffraction pattern for a typical (110) surface with the simulated pattern and Miller indices in (d).

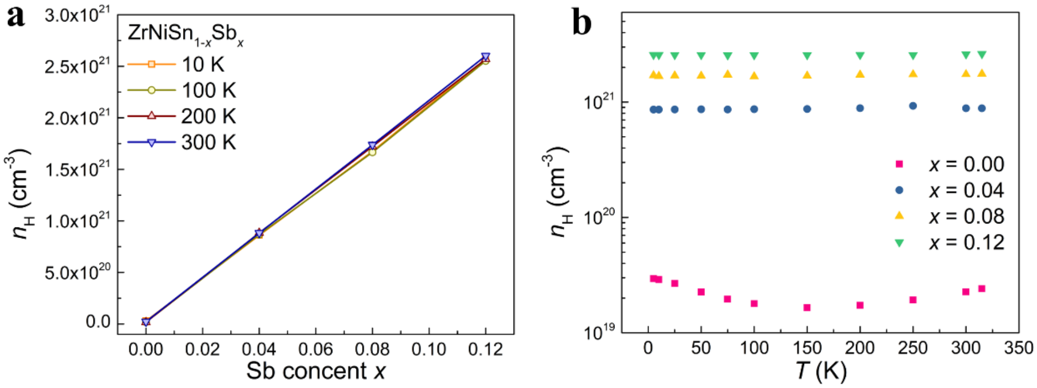

Supplementary Figure 3 Hall carrier concentration n_H. (a) n_H as a function of Sb content at 10 K, 100 K, 200 K and 300 K, (b) n_H as a function of temperature for $ZrNiSn_{1-x}Sb_x$ over the temperature range of 5 K to 315 K. The almost T-independent n_H indicates that the ions should be fully ionized below 5 K. This excludes the effect of increased n_H on the discussion of carrier mobility in Figure 2.

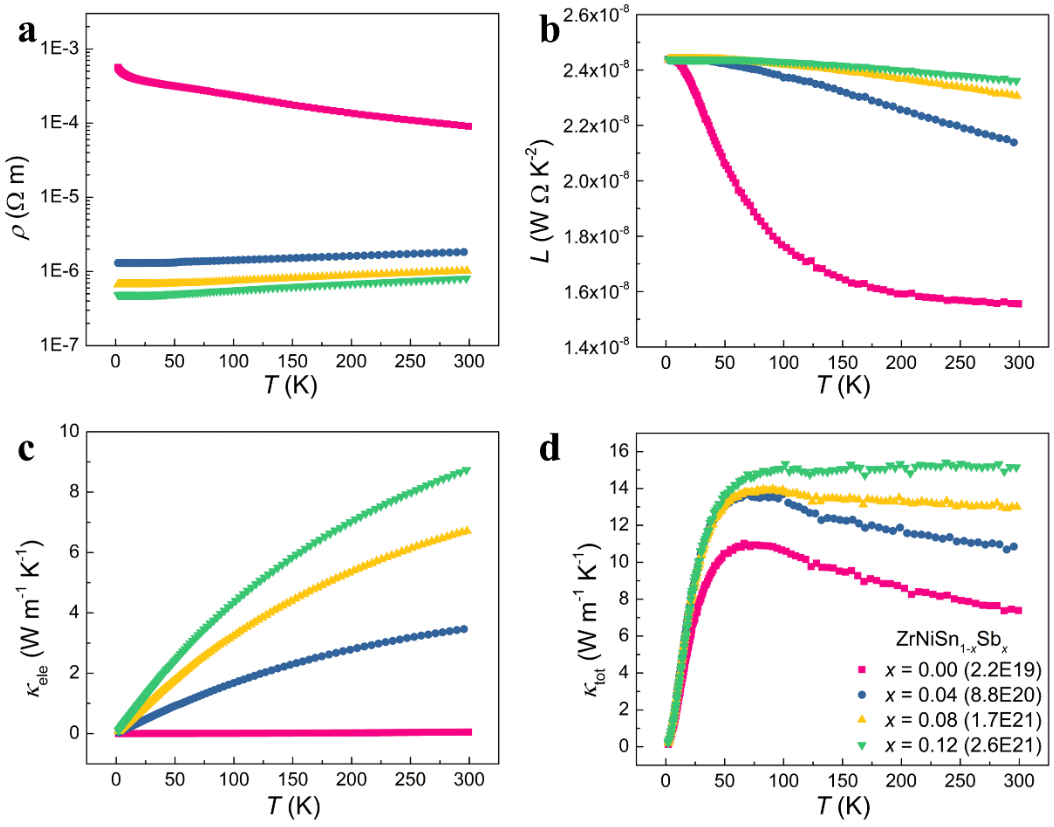

Supplementary Figure 4 Transport properties as functions of temperature. (a) Electronic resistivity ρ, (b) Lorenz number L, (c) calculated electronic contribution to thermal conductivity κ_{ele}, (d) measured total thermal conductivity κ_{tot}. The κ_{ele} is estimated with the Wiedemann–Franz law, $\kappa_{ele} = LT/\rho$.

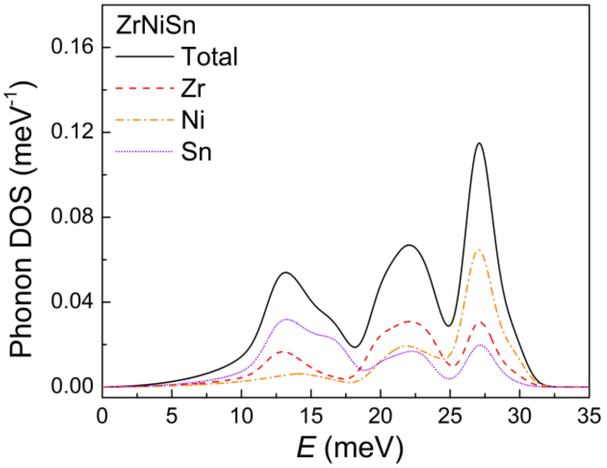

Supplementary Figure 5 Calculated phonon density of states (DOSs). (a) Total and partial phonon DOS, without considering difference of atomic mass, m, and neutron scattering cross section, σ, between different elements, for ZrNiSn obtained from first-principles calculations. The neutron-weighted phonon DOS for ZrNiSn are shown in Figure 3(c) in the main context. In inelastic neutron scattering measurements for $ZrNiSn_{1-x}Sb_x$ compounds, the contribution from Ni element is overemphasized: $g_{NW}(E) = \left[\frac{\sigma_{Zr}}{m_{Zr}} g_{Zr}(E) + \frac{\sigma_{Ni}}{m_{Ni}} g_{Ni}(E) + (1-x)\frac{\sigma_{Sn}}{m_{Sn}} g_{Sn}(E) + x\frac{\sigma_{Sb}}{m_{Sb}} g_{Sb}(E)\right]/3$, where $g_{Zr}(E)$, $g_{Ni}(E)$, $g_{Sn}(E)$ and $g_{Sb}(E)$ are the partial densities of states of Zr, Ni, Sn and Sb, respectively. The values of σ/m for (Zr, Ni, Sn, Sb) are (0.07081, 0.31520, 0.03471, 0.03203) b per amu, respectively.

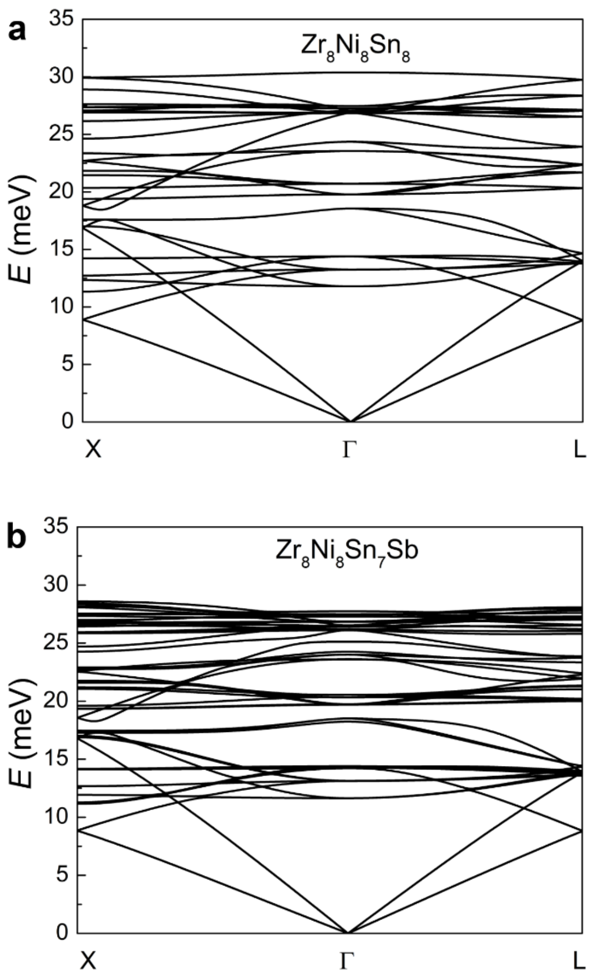

Supplementary Figure 6 First-principles calculation of the phonon dispersions in the supercell (a) ZrNiSn (or $Zr_8Ni_8Sn_8$) and (b) $ZrNiSn_{0.875}Sb_{0.125}$ (or $Zr_8Ni_8Sn_7Sb$). In contrast to the obvious LO-TO splitting in $Zr_8Ni_8Sn_8$, doping of Sb on Sn site breakdown this splitting due to increased screening effect.

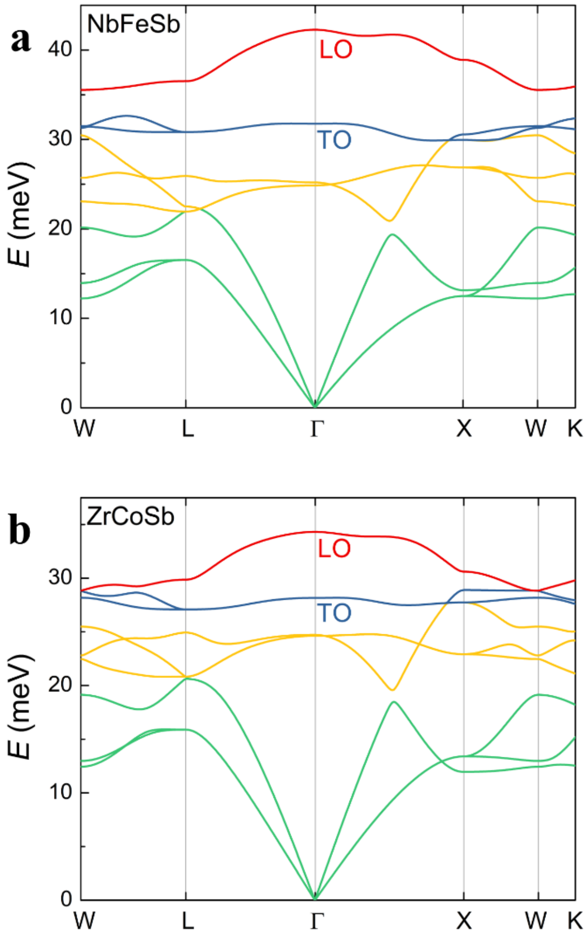

Supplementary Figure 7 First-principle calculation of the phonon dispersions for (a) NbFeSb and (b) ZrCoSb. Both samples exhibit obvious LO-TO splitting.

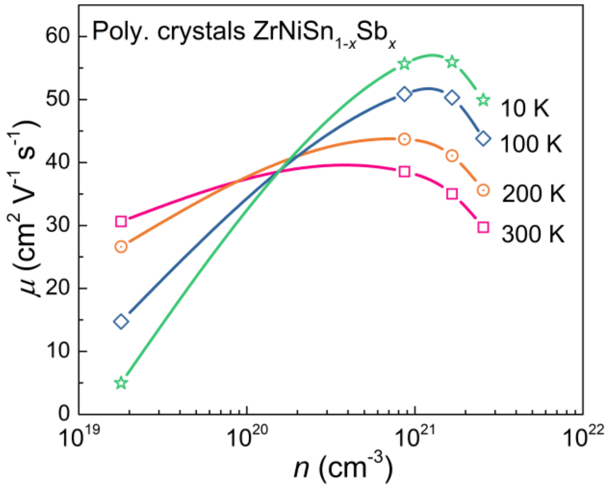

Supplementary Figure 8 Carrier mobility as a function of carrier concentration *n* in polycrystalline $ZrNiSn_{1-x}Sb_x$ samples at 10 K, 100 K, 200 K and 300 K. The solid lines are guides of the eye.

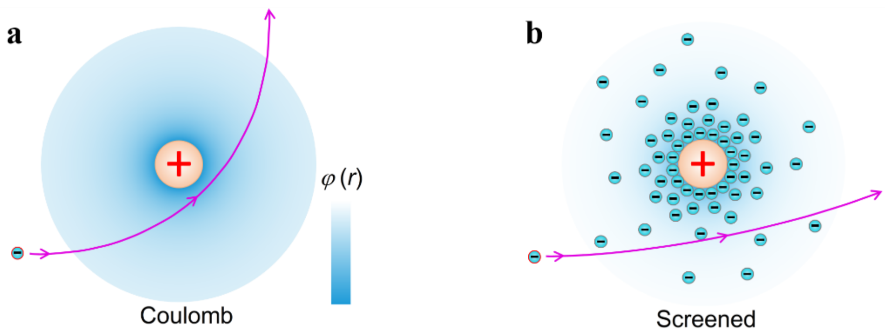

Supplementary Figure 9 Schematic demonstration of the screened ionized impurity scattering. (a) Bare Coulomb potential of an ionized impurity center ($\varphi(r) \propto \frac{1}{r}$) as a function of distance r in real space and (b) screened potentials ($\varphi(r) \propto \frac{1}{r}\exp(-\frac{r}{r_{\mathrm{TF}}})$) by free carriers in the approximation of Thomas-Fermi model. Compared with the bare Coulomb potential, the screened potential drops more quickly with distance, and the momentums of traveling charge carriers are less changed when they pass through the ionized impurity scattering centers.

Supplementary Table 1 The polar coupling constant for ZrNiSn, NbFeSb and ZrCoSb.

**Material**	$\boldsymbol{\omega_{LO}/\omega_{TO}}$	$\boldsymbol{\varepsilon_{\infty}}$	$\boldsymbol{\varepsilon_{s}}$	$\boldsymbol{\hbar\omega_{LO}}$	$\boldsymbol{m^*}$ (m_e)	$\boldsymbol{\alpha_{PO}}$
ZrNiSn	1.243	22.072	27.435	30.328	2.8	0.32
NbFeSb	1.771	27.475	48.659	42.273	6.9	0.74
ZrCoSb	1.485	18.628	27.661	34.312	6.5	0.89

The parameters, ω_{LO}, ω_{TO}, ε_{∞}, ε_{s} and $\hbar\omega_{LO}$, are determined from the first-principle calculations, and m^* are cited elsewhere.[1, 2, 3] The phonon dispersions for NbFeSb and ZrCoSb are shown in Supplementary Figure 7, which show obvious LO-TO splitting.

References:

1. Xie H, *et al.* The intrinsic disorder related alloy scattering in ZrNiSn half-Heusler thermoelectric materials. *Sci. Rep.* **4**, 6888 (2014).

2. Qiu Q, *et al.* Grain Boundary Scattering of Charge Transport in n-Type (Hf,Zr)CoSb Half-Heusler Thermoelectric Materials. *Adv. Energy Mater.* **9**, 1803447 (2019).

3. Fu C, *et al.* Realizing high figure of merit in heavy-band p-type half-Heusler thermoelectric materials. *Nat. Commun.* **6**, 8144 (2015).
